# Answering a four decade-old question on epicuticular wax biosynthesis

**DOI:** 10.1093/jxb/erw144

**Published:** 2016-05-07

**Authors:** Dylan K. Kosma, Owen Rowland

**Affiliations:** ^1^Department of Biochemistry and Molecular Biology, University of Nevada Reno, Reno, Nevada, 89557, USA; ^2^Department of Biology and Institute of Biochemistry, Carleton University, Ottawa, Ontario, K1S 5B6, Canada

**Keywords:** Barley (*Hordeum vulgare*), β-diketone aliphatics, carboxylesterase, *Cer-cqu* gene cluster, cytochrome P450, diketone synthase (DKS), epicuticular wax, esterified alkan-2-ols, hydroxy-β-diketones, lipase, type III polyketide synthase (PKS).

**In this issue of *Journal of Experimental Botany* (pages 2715–2730) Schneider *et al.* report the identity of three genes from barley described in the 1970s as important for the synthesis of β-diketone cuticular waxes, thereby revealing a novel polyketide synthase pathway responsible for their production. It is a perfect example of how modern sequencing technologies can resolve age-old questions on important food crops.**

All land plants possess a lipophilic layer coating their aerial surfaces: a cuticle. It consists of waxes embedded within and overlaying an esterified polymer of oxygenated fatty acids and glycerol (cutin). As such, plant cuticles form one of the largest biological interfaces on the planet, providing the first points of contact with the surrounding, often hostile, environment. The key function of cuticle is to prevent non-stomatal water loss, but other functions include defense against bacterial and fungal pathogens, mediating interactions with insects, and protection from excess levels of ultraviolet radiation.

Cuticular waxes are chemically complex mixtures of hydrophobic molecules, most typically even and odd chain fatty acid derivatives of carbon lengths C_24_ and higher. Epicuticular waxes form the outermost layer of cuticle and often form crystals, imparting a whitish bloom to the organ surface. The amount and composition of cuticular wax varies widely between plant species and even between organs of the same plant; and cuticle chemistry can also change during development of the organ and is altered by environmental conditions.

β-diketones are very-long-chain (typically C_29_–C_31_) oxygenated hydrocarbons that have long been known to be cuticular wax components of some plants (Horn and Lamberton, 1962; Tulloch and Weenink, 1966; Jackson, 1971; von Wettstein-Knowles, 1972; Evans *et al.*, 1975; Jenks *et al.*, 2002). They are well described in the cuticular waxes of diverse plant species, including *Eucalyptus*, *Rhododendron* and *Hosta*. However, they are best described in graminaceous species (e.g. barley, wheat and oats) (Box 1). Knowledge of β-diketone biosynthesis has come from extensive genetic and radiotracer feeding studies in barley by Penny von Wettstein-Knowles and colleagues (von Wettstein-Knowles, 1995; 2012). The largest collection of cuticular wax-deficient mutants, termed *eceriferum* (*cer*), is in barley, consisting of more than 1500 mutants in about 75 complementation groups. The *Cer-cqu* cluster, which affects β-diketone, hydroxy β-diketone and esterified alkan-2-ol production, is represented by over 500 distinct mutations and is made up of three complementation groups (*Cer*-*c,* -*q* and -*u*) (Box 1). The identities of the genes in this locus have remained elusive – until now.

**Box 1. Epicuticular waxes of barley flag leaf sheaths**

**Figure:**
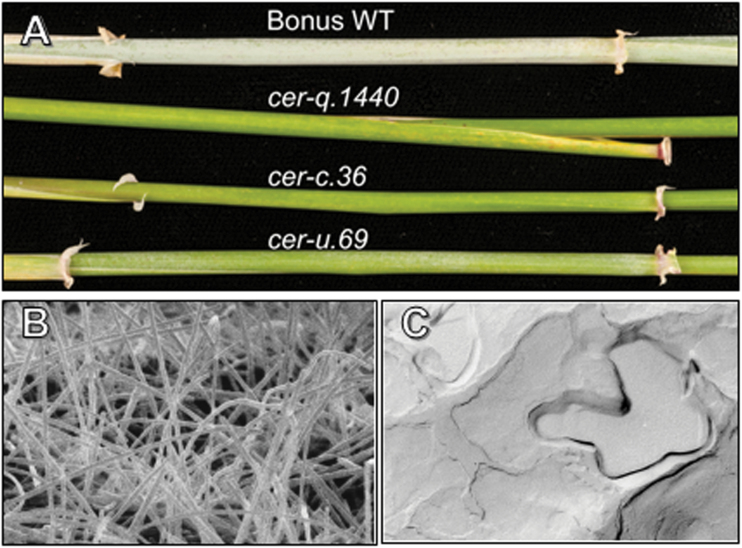
(A) Wild type (WT) barley ‘Bonus’ and *cer-c*, *cer-q*, and *cer-u* mutants demonstrating the glossy phenotypes of the *cer-c* and *cer-q* mutants plus reduced wax on the *cer-u* mutant resulting from reductions in β-diketones and their derivatives. (B) Scanning electron micrograph image from Bonus. The long, hollow tubular structures can be attributed to large quantities of β-diketone aliphatics. (C) Transmission electron micrograph image of a pre-shadowed carbon replica from *cer-c.36* revealing flat plates of wax in the absence of β-diketone tubes. Images are courtesy of and reproduced with permission from Nikolai M. Adamski and Penny von Wettstein-Knowles.

## Significant new evidence

The canonical pathway for wax synthesis involves the export of fatty acids from the chloroplast, elongation of fatty acyl-CoAs by endoplasmic reticulum-localized fatty acid elongase complexes, and subsequent modification of elongated acyl-CoAs to primary alcohols, alkyl esters, aldehydes, alkanes, secondary alcohols, ketones and free fatty acids. The genes encoding the enzymes, transporters and regulators for these aliphatics have been revealed in Arabidopsis using its extensive genetic resources (Samuels *et al.*, 2008). However, Arabidopsis does not produce β-diketone cuticular waxes and is thus unsuitable for deducing the synthesis of these compounds.

β-diketones and their hydroxylated derivatives have been hypothesized to be synthesized and elongated by a β-ketoacyl elongase system (Netting and Wettstein-Knowles, 1976; Mikkelsen, 1979; 1984). However, this work by Schneider *et al.* (2016) presents evidence validating a model presented more recently (von Wettstein-Knowles, 2012) that β-diketones are synthesized by a polyketide-like pathway. More explicitly, the identification of CER-C as a chalcone-synthase-like diketone synthase (DKS) from the type III polyketide synthase superfamily provides strong evidence that β-diketone synthesis is achieved by only two condensations catalyzed by DKS, instead of the three condensations typical of chalcone synthases for the production of flavonoids and other similar molecules. Notably, this chalcone synthase-like DKS enzyme appears only to catalyze linear condensation and elongation reactions and not also the cyclization reactions typical of other type III polyketide synthases.

The identification of CER-U as a cytochrome P450 hydroxylase fits perfectly with the reduced amounts of hydroxy β-diketones in the waxes of *cer-u* mutants. CER-U most likely possesses in-chain hydroxylase activity. CYP450s with in-chain hydroxylase activities on aliphatics have been described (Kandel *et al.*, 2005; Greer *et al.*, 2007). All evidence points to C_29_ and C_31_ β-diketones as the native substrates of CER-U, although this remains to be confirmed biochemically.

The annotated function of the *Cer-q* gene as encoding a lipase/carboxylesterase is perplexing. A strong body of evidence implicates CER-Q as preceding the reactions that lead to both β-diketones and alkan-2-ols (Netting and von Wettstein-Knowles, 1976; Mikkelsen, 1984). The model proposed by Schneider *et al.* (2016) suggests that CER-Q plays a role in cleaving acyl chains destined to be alkan-2-ols and β-diketones from a glycerolipid. A thorough biochemical characterization of the enzyme encoded by the *Cer-q* gene will clarify the precise role of this ‘lipase/carboxylesterase’ in the biosynthesis of alkan-2-ols and β-diketones.

Another significant finding presented by Schneider *et al.* (2016) is that *Cer-c, -q,* and *-u* represent three distinct genes encoding separate proteins instead of what was previously predicted to be a single gene, *Cer-cqu*, encoding a multifunctional polypeptide. This brings to light important questions about the association of proteins into multienzyme complexes to form metabolons, which could be particularly important for improving the efficiency of enzymatic modification of aliphatics in the predominantly aqueous environment of the cell including, potentially, β-diketones and hydroxy β-diketones.

## Enigmatic genes explained?

After more than 40 years of study, the genes underlying the *Cer-cqu* locus have finally been identified. The predicted functions of CER-C and CER-U from studies conducted in the 1970s by Mikkelsen and von Wettstein Knowles are very much corroborated by the identification and annotated functions of the genes associated with these loci. The biochemistry to validate enzyme activities of these gene products will be an exciting endeavor.

Several questions remain to be answered. The mechanism of further elongation beyond the CER-C/DKS-catalyzed condensations necessary to obtain the very-long-chain lengths of cuticular β-diketones (C_29_ and C_31_ in barley) remains unknown. Logically, a fatty acyl elongase (FAE)-based elongation is probable, but whether CER-C/DKS can associate with FAE complexes remains unknown and will be an interesting question to pursue with the advanced cell biology tools that are available today. The role of CER-Q in β-diketone and alkan-2-ol biosynthesis remains enigmatic. Biochemical investigation of this protein will likely facilitate the discovery of novel biochemical steps required for plant cuticular wax synthesis.

The introduction of the *Cer-cqu* locus gene cluster into heterologous systems will be an interesting approach for determining whether β-diketones can be produced in other plant species, their effect on wax crystallization patterns, and their effects on cuticle function. The glaucousness, or bluish-grey wax bloom, of cereal crops has been associated with desirable agronomic traits like grain yield and drought tolerance (Kosma and Jenks, 2007). The lack of β-diketones in many of the *cer* mutants results in a ‘glossy’ or non-glaucous phenotype (Box 1). Thus, the *Cer-cqu* gene cluster stands to be of potential significance for crop improvement.

This paper by Schneider *et al.* (2016) answers a question that has persisted for four decades and has come full circle to test hypotheses presented in the 1970s about these enigmatic genes. However, rather than drawing this research to a conclusion, this discovery facilitates years of future research on the biochemistry of these fascinating enzymes.
